# The Use of the ATD Technique to Measure the Gelation Time of Epoxy Resins

**DOI:** 10.3390/ma14206022

**Published:** 2021-10-13

**Authors:** Jakub Smoleń, Piotr Olesik, Paweł Gradoń, Mateusz Chudy, Bogusław Mendala, Mateusz Kozioł

**Affiliations:** 1 Faculty of Materials Engineering, Silesian University of Technology, Krasińskiego 8 Street, 40-019 Katowice, Poland; piotr.olesik@polsl.pl (P.O.); pawel.gradon@polsl.pl (P.G.); boguslaw.mendala@polsl.pl (B.M.); mateusz.koziol@polsl.pl (M.K.); 2 Faculty of Mechanical Engineering, Silesian University of Technology, Konarskiego 18A Street, 44-100 Gliwice, Poland; matechu706@student.polsl.pl

**Keywords:** polymer resin, analysis of temperature derivative (ATD), polymer matrix composite, graphite, carbon fiber

## Abstract

In this paper, we investigated the thermodynamics of the resin curing process, when it was a part of composition with graphite powder and cut carbon fibers, to precisely determine the time and temperature of gelation. The material for the research is a set of commercial epoxy resins with a gelation time not exceeding 100 min. The curing process was characterized for the neat resins and for resins with 10% by weight of flake graphite and cut carbon fibers. The results recorded in the analysis of temperature derivative (ATD) method unequivocally showed that the largest first derivative registered during the test is the gel point of the resin. The innovative approach to measuring the gelation time of resins facilitates the measurements while ensuring the stability of the curing process compared to the normative tests that introduce mechanical interaction. In addition, it was found during the research that the introduction of 10% by weight of carbon particles in the form of graphite and cut carbon fibers rather shortens the gelation time and lowers the temperature peak due to the effective absorption and storage of heat from the cross-linking system. The inhibiting (or accelerating) action of fillers is probably dependent on chemical activity of the cross-linking system.

## 1. Introduction

The use of epoxy resins in polymer matrix composites (PMC) is dictated by gaining good properties, especially in terms of mechanical strength, electrical insulating properties, and resistance to environmental and chemical corrosion [1]. The PMCs are commonly used in many industries due to relatively easy processing methods. Introducing the reinforcement material to the volume of the resin leads to the improvement of certain properties [2,3,4,5] but may cause difficulties, e.g., sedimentation of powder fillers leading to inhomogeneity of the material. Depending on the morphology of filler, there may be different interactions between the matrix phase and the reinforcement phase [6,7,8]. Spherical filler interacts with the resin matrix in a different way than fibers, for which the interaction depends on the length of the fibers and their orientation/arrangement. The introduction of fillers into polymer composites is a complex issue that closely depends on the type of liquid matrix. It is generally accepted that thermoplastic polymers are more difficult to incorporate powder fillers than thermosetting polymers due to the significantly higher viscosity in the processing state [9,10].

The important parameters describing the resins are the pot life and the gelation time. The pot life of the resin is the time after which the use of the resin (composed with hardener or catalyst) in the technological process becomes impossible due to high viscosity. The gelation time is the time it takes for the resin to go from sol to gel. The precise determination of the gelation time is difficult due to the complex nature of the phenomena during cross-linking of the resin. So far, numerous methods for determining the gelation time have been developed, which ensures reproducible results, and their accuracy varies. The most widely described methods of measuring the gelation time of resins can be found in the review article by A.A. Shimkin [11], where the author describes 28 different measurement methods, considering their limitations and possibilities. The author of the publication draws attention to the basic problems in the precise determination of the gelation time of resins resulting from the gradual transition of a viscous liquid to an elastic solid. Among many normative tests, the dominant group are manual methods [12,13,14]. Manual methods are not the simplest measuring methods and require extensive knowledge of resins and curing mechanisms. The main problem is the lack of reproducibility of the results resulting from the experience of the evaluator. Despite the low precision of the results, these methods are popular due to their low costs and requiring no expensive and complicated research equipment. Currently, manual methods are replaced by automatic measurement methods due to their simplicity and repeatability. Automatic methods, like manual methods, are subject to standardization [15,16,17,18], and there are more and more patents for original design solutions for measuring devices.

The use of a thermal scanning rheometer (TSR) to determine the gelation time [19,20] is effective and enables two alternative methods of measurement. The first method is to draw the tangent at the yield point on the G′ curve. The point at which the drawn tangent intersects with the baseline is assumed as the gelation time. This is the point where the fabric shows elasticity. The second calculation method assumes the determination of the point at the intersection of the G′ and G″ curves. The intersection of the curves satisfies the criterion of equality between the energy lost and the stored energy. At the point of intersection of the curves, the material is both viscous and elastic. The results of both methods are similar and differ by a maximum of about 10%. A wide group of methods for measuring the gelation time are methods based on dynamic mechanical analysis (DMA), where a particularly important parameter is the viscoelastic nature of the material. The gelation time is determined by extrapolating the onset of growth of the modulus of elasticity or the maximum value of the energy loss tangent [21,22,23]. Dielectric analysis (DEA) methods focus on measuring the polarization of the resin in an alternating electric field. The limitation of the DEA methods is the limited thickness of the sample and their polar character [20,24]. In addition, use of differential scanning calorimetry (DSC) for gel time determination is popular. In this method, two samples of resin are measured: fresh resin after mixing with hardener and resin quenched just after gelation. By comparing their reaction heat (ΔH), the degree of conversion (α) vs. time plot can be obtained. The gel time is the moment of achieving α of quenched sample of freshly prepared resin [25,26,27]. The last methods of measuring gelation time, often described in the literature, are ultrasonic methods [28,29]. Gel time is measured based on high frequency (above 20,000 Hz) mechanical vibration damping.

When determining the rate of curing of the resin, many variables must be considered. After adding the hardener, the resin cross-linking process begins, the course of which depends on the chemical structure of the resin and the hardener, their quantitative ratios, the volume of the mixture, and physical conditions, e.g., temperature and pressure [30,31]. The volume and morphology of the reinforcement phase also have a significant influence on the curing rate. The exothermic cross-linking reaction initiated thermally is dependent on the heat of reaction between the resin and the hardener. As the amount of resin increases, the efficiency of heat removal from the hardened system decreases, which causes a significant increase in the temperature of the system and shortens the life of the composition. Therefore, when conducting research on the cross-linking process, it is important to maintain a constant volume of the mixture. Large volumes of mixtures can lead to local overheating of the composite and the introduction of internal stresses, which, in extreme cases, lead to cracking of the casting [32,33,34]. The control of the curing temperature significantly influences the cross-linking rate, which is described by the Arrhenius law, where lowering the temperature by 10 °C causes a two-fold decrease in cross-linking rate [35,36,37,38]. In an industrial condition, measuring the rise in temperature is more important than measuring the overall thermal effect of a reaction. The temperature course enables the curing rate to be determined, depending on the reactivity of the resin with the hardener and on the curing temperature.

The ATD (analysis of temperature derivative) method is principally used in metal alloy testing for determination of temperature of phase changes under free cooling conditions. This method consists of submerging a thermocouple of certain type in the cooling alloy. The temperature of alloy is continuously registered in the desired range (typically until complete solidification), and the first-time derivative of temperature is calculated from this data. The results of ATD are generally presented as a cooling curve with superimposed derivative curve. The cooling rate of alloy is affected by released latent heat of phase transitions. These changes can be seen directly on the temperature curve, but the determination of exact values of phase change temperature can be difficult, and so the derivative curve is used instead. Many different methodologies were developed for interpretation of ATD curves in response to different needs in metal alloys analysis. The choice of methodology depends on type of alloy (steel, cast iron, aluminum, superalloys, etc.) or on desired effect of analysis (scientific investigation, quality control, chemical composition analysis, etc.) [39,40,41,42].

This article describes the thermal analysis ATD of curing processes in commercial epoxy resins and their compositions with the addition of flake graphite and cut (milled) carbon fibers. Comparison of the results for the unfilled epoxy resin with the results for resins with the addition of carbon fillers will allow to compare the thermal effects of curing processes and describe their effect on the rate and temperature of curing. Measurement of the gelation time using the thermal derivative analysis (ATD) method was performed. The determination of the time and temperature of gelation using the ATD method is an innovative solution that allows obtaining precise measurement results in an automated and repeatable manner. The study refers to the earlier research conducted on curable polymer resins and concerned with the effect of volume on the intensity of curing process [32,33,34].

## 2. Materials and Methods

### 2.1. Materials

For the purposes of the study, a set of commercial epoxy resins with dedicated hardeners was prepared, as is given in Table 1. The reinforcing phase of the compositions used in the tests was alternative carbon filler added in the amount of 10% by weight. The first filler was flake graphite Grafit-390 (Biomus, Lublin, Poland), with a mesh size of 325. The second one was milled carbon fiber SIGRAFIL^®^ C M150-4.0/240-UN394-150 (SGL TECHNOLOGIES GmbH, Meitingen, Germany), with an average length of 150 µm (range 135–155 µm).

### 2.2. Sample Prepration

The test procedure consisted of adding the resin and the hardener in stoichiometric ratios by weight so that their sum was constant and always equal to 100 g. A total of 10 g of graphite/ground carbon fibers was added to the resin mixture and homogenized until the mixture was homogeneous. After about 3 min of mixing, the hardener was added to the system and the gelation time measurement was started. The results obtained with the standard-based method were compared with the results of ATD measurements.

### 2.3. Gelation Time Measurement with Standard-Basedmethod

The gelation time for selected resins was measured according to DIN 16945 [18]. Samples of resin mixed with hardener was placed in polypropylene cup in amount of 100 g. At interval of 30 s, from resin string of liquid was drawn with the wooden probe. The gelation time has been determined as a time to the moment when the 20-mm-long continuous “threads” were pulled from the resin. The test was carried out at room temperature 25 °C.

### 2.4. Thermal Derivative Analysis Measurement

ATD analysis was carried out using type K (chromel–alumel) thermocouples and the Z-TECH Crystaldigraph-PC-8T converter (produced by Z-TECH, Gliwice, Poland) coupled with a PC-type computer using Z-TECH MLab2 1.2 analysis software (by Z-TECH, Gliwice, Poland) [43]. Thermocouples were prepared with twisted junction and submerged directly in the resin mixture, without any cover, to minimize thermal inertia. In the testing setup, the measuring ends of thermocouple were placed near the center of the volume of resin containers (concerning all three dimensions). The test was carried out with 50 g resin sample at room temperature, 25 °C.

### 2.5. Gel Time Determination from ATD

**Figure 1:**
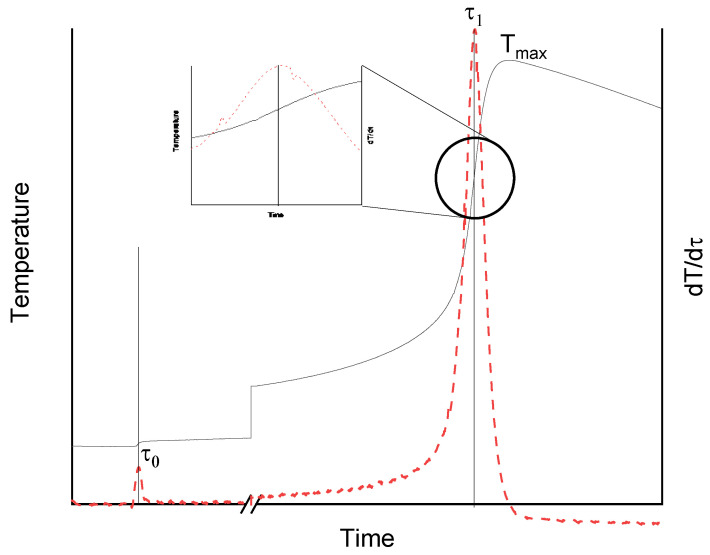
Sample curve of resin gelation process by ATD method: solid line—resin temperature vs. time; dotted line—1st derivative of resin temperature vs. time curve; τ_0_—time of thermocouple insertion into resin; τ_1_—gelation time of resin.

The data obtained from ATD were processed with Origin 2019 (version 9.6.5.169, Origin Lab Corporation, Northampton, MA, USA). The derivatives were calculated using Origin base functions and obtained curves were smoothed by Savitzky–Golay method. Sample curve is presented in Figure 1, where the gelation phenomena is indicated by peak τ_1_. To calculate gelation time of resin, the Equation (1) was used.

(1) $$\tau_{\mathrm{gel}}=\frac{\tau_{1}-\tau_{0}}{60}$$

where τ_gel_—gelation time of resin (min), τ_0_—time of thermocouple insertion into resin (s), and τ_1_–time of resin gelation (s).

## 3. Results and Discussion

### 3.1. ATD vs. Standard-Based Method

**Figure 2:**
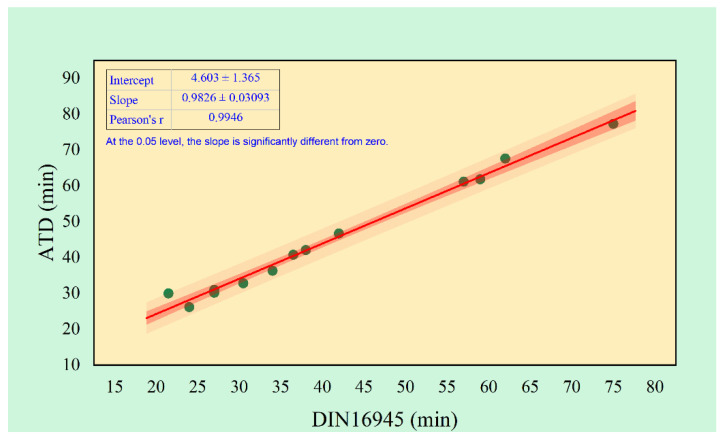
Standard-based method vs. ATD correlation and regression results.

The results of comparative verification of two methods applied for determining gelation time of investigated resins are presented in Table 2. To validate ATD method, correlation and regression analysis against standard-base determined values was performed. The regression curve is presented in Figure 2. Obtained Pearson’s *r* coefficient has a value 0.9999, which means there is strong correlation between the two applied methods. In addition, all experimental points are in the prediction band. Such results indicate that ATD method is a valid tool for resins gelation time determination. However, it should be noticed that non-isothermal resin measurements are sensitive for ambient temperature changes or even small variation of hardener–resin ratio.

The results in Table 2 have been achieved during curing of the individual resins in accordance with the technical recommendations of the manufacturers, without additional inhibitors or accelerators. Importantly, both standard-based and ATD trials were performed in the same room and at the same time. The cross-linking conditions of the resins were therefore comparable.

### 3.2. Gelation Time Measurement with ATD

**Figure 3:**
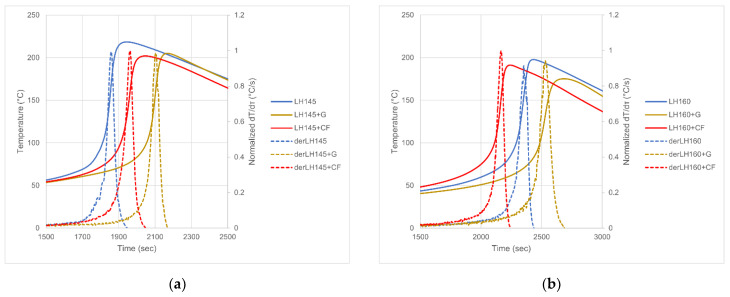

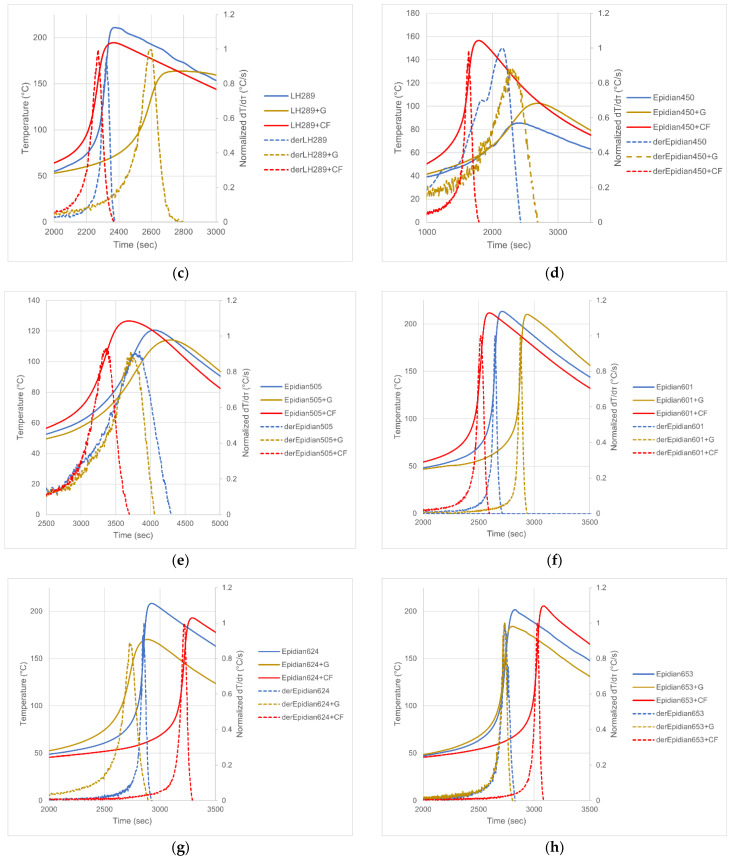

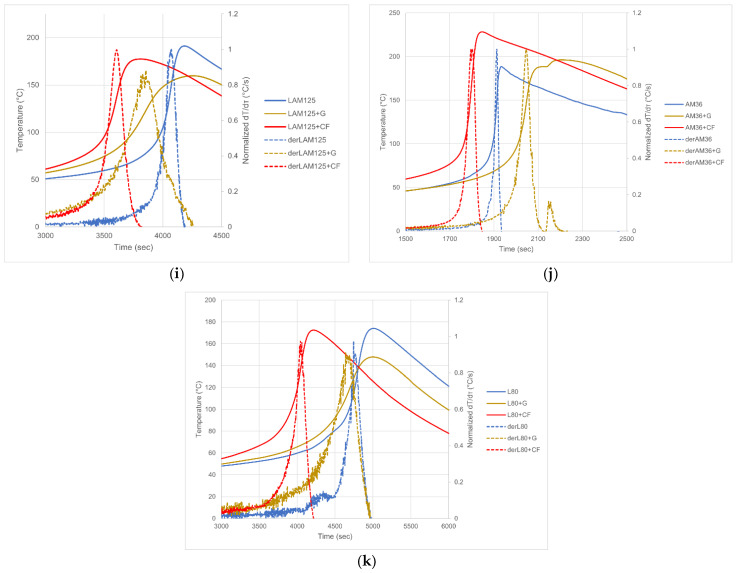
ATD curves of investigated resins: (**a**) LH145, (**b**) LH160, (**c**) LH289, (**d**) Epidian450, (**e**) Epidian505, (**f**) Epidian601, (**g**) Epidian624, (**h**) Epidian653, (**i**) LAM125, (**j**) AM36, (**k**) L80.

The results of ATD gelation time measurement are presented in Figure 3, and the values of gelation time and maximum curing temperature are summarized in Table 3. The curves of derivatives are normalized to the gelation peak value to clearly indicate the second-order phase transition at curing curves. The gelation process was described as continuous phase transition by Rouwhorst et al. [44], and our results correspond with their observation well. It can be observed that for some resins the introduction of graphite or carbon fibers has significantly changed the curing process.

In most of the studied cases, the addition of carbon fibers or graphite caused a drop in peak temperature and an extension of gelation time, but in some cases, the effect is opposite. The influence of carbon additives on the resin cross-linking process will result from the simultaneous occurrence of two effects:

(1) Catalytic surface action of carbon additives. Both active groups on the surface of fibers and graphite, formed in pyrolysis processes, and molecules (mainly oxygen) adsorbed on the surface of the additives are responsible for the effect. Catalytic effects of various types of carbon additives on polymer matrices are described, inter alia, in works [45,46,47]. Catalytic reactions of fillers are also often occurring in case of phosphoric flame retardants [48,49]. The catalytic effect stimulates **accelerating** the resin cross-linking process. It should result in an increase in peak temperature and a shortening of gelation time.
(2) Local heat absorption from the area surrounding the particle (or fiber). It results from the very good thermal conductivity of graphite and carbon fibers (which is mostly also made of graphite) and the relatively high heat capacity of such particles or fibers. This effect **inhibits** the resin cross-linking process. It should result in lowering peak temperature and extending gelation time.

For some of the resins tested, the former effect is more intense than the latter, and for others, the opposite is true. All the data are summarized in Table 3.

The resins, for which the addition of fibers and graphite decreased peak temperature and extended gelation time, were LH145, LH160, and LH289—all “LH” resins. The trend is clear here. The common feature of the resins is the relatively high average epoxy number (AEN), amounting to 0.535–0.575 mol/100 g (values from the technical documentation of Havel Composites [50]).

The behavior of “Ep” type resins, on the other hand, is ambiguous. In their case, the behavior under the influence of the addition of graphite and carbon fiber changes from acceleration to inhibition with the increase of the average epoxy number (AEN) of the resin: Ep450—acceleration (AEN = 0.35 mol/100 g); Ep505—“hybrid” behavior, acceleration with fibers, inhibition with graphite (AEN = 0.4 mol/100 g); Ep601 and Ep653—”neutral” behavior, no inhibition and no acceleration noticed (AEN = 0.525 mol/100 g); Ep624—evident inhibition (AEN = 0.5 mol/100 g and addition of chemically active thinner). The given values of epoxy numbers were taken from the catalog of the resin producer [51]. Increasing the number of chemically active groups involved in the cross-linking reactions causes a more inhibitory effect of carbon additives during the cross-linking process. This can be explained by the fact that the amount of active/adsorbed active media on the surface of the particles is limited, and the concentration of active chemical groups on the side of the resin/hardener/thinner neutralizes their influence. In such a situation, the inhibitory effect due to the local absorption and storage of the heat of reaction by the particles prevails.

We have not been able to obtain data on the AEN of LAM125, AM36, and L80 resins; this is a declared secret of the manufacturers. However, it can be expected that the LAM125 and L80 resins will have a relatively high AEN; they are aviation approved resins, so they must exhibit reproducible high strength and modulus of elasticity. These two characteristics usually grow with AEN. The AM36 resin, on the other hand, is more for general use. Therefore, it can be expected that the results presented in Table 3 confirm the trend obtained for the Ep and LH resins—LAM125 and L80 as resins with probably higher AEN are subject to inhibition due to added particles, while the AM36 resin, assuming its lower AEN, is accelerated after adding particles in the cross-linking process. The observed trend of the action of carbon particles depending on AEN requires further research, but the obtained results clearly indicate that this type of research should be carried out.

As for the comparison of the obtained results with the action of a particular type of particles, it can be stated that graphite causes a more intense inhibitory effect, and the fibers act more active during accelerate direction. Perhaps graphite is more dispersed in the mass of resin, consists of smaller particles than fiber, has a larger specific surface area. As a result, it absorbs heat better (same heat capacity, but faster absorption due to the larger surface area). On the other hand, there are probably more catalytic centers formed during pyrolysis on the fibers—as a rule, the precursors of carbon fibers are polyacrylonitrile or petroleum pitch, containing numerous chemical components that may remain (in their original or thermally changed form) on the surface of the fibers after pyrolysis.

**Figure 4:**
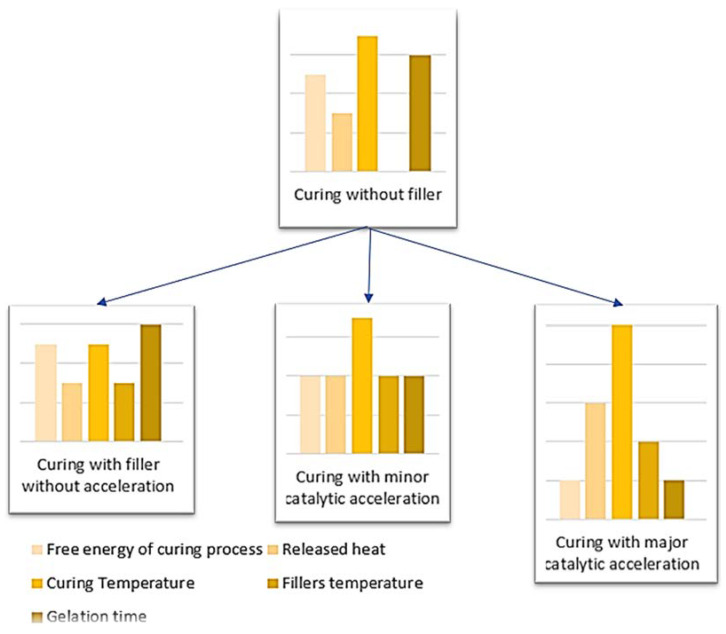
Schematic comparison of released heat in resins with fillers.

The schematic model of heat released during curing process is presented in Figure 4.

## 4. Conclusions

1. Temperature vs. time relation for resin curing process reflects the second-order phase transition, and specific changes within this relation may be characterized by 1st derivative;
2. The Analysis of Temperature Derivative is a valid tool for determination of resin’s gelation time;
3. The proposed method can be easily adapted into automatic measurement procedures with high resolution;
4. The addition of graphite particles or milled carbon fibers to resins significantly and ambiguously affects gelation time and peak temperature of curing process in most of tested samples; and
5. Increasing the number of chemically active groups involved in the reactions of the cross-linking process—including epoxy groups—probably causes a more inhibitory effect of carbon additives (graphite particles of milled carbon fibers). This effect should be further and more widely investigated to confirm it.

## Figures and Tables

**Table 1:** Commercial epoxy resins and hardeners used in the research.

Sample Name	Epoxy Resin	Hardener	Weight Ratio (g)
LH 145	LH 145 ^1^	H 135 ^1^	100:35
LH 160	LH 160 ^1^	H 135 ^1^	100:35
LH 289	LH 289 ^1^	H 135 ^1^	100:35
Ep450	Epidian^®^ 450 ^2^	Z-1 ^2^	100:8.4
Ep505	Epidian^®^ 505 ^2^	Z-1 ^2^	100:10
Ep601	Epidian^®^ 601 ^2^	Z-1 ^2^	100:12.6
Ep624	Epidian^®^ 624 ^2^	Z-1 ^2^	100:11.8
Ep653	Epidian^®^ 653 ^2^	Z-1 ^2^	100:12.3
LAM125	LAM-125 ^3^	LAM-226 ^3^	100:30
AM36	Ampreg™ 36 ^4^	Ampreg™ 3X Fast ^4^	100:29
L80	C-L L80 ^5^	LH 55 ^5^	100:30

^1^ Havel Composites (Svesedlice, Czech Republic); ^2^ CIECH S.A. (NowaSarzyna, Poland); ^3^ PRO-SET (Bay City, MI, USA); ^4^ GURIT (Wattwill, Switzerland); ^5^ C-L Sp. z o.o. (Słupsk, Poland).

**Table 2:** Results of gelation time for standard-based method and ATD method.

Sample	Standard Method (min)	ATD Method (min)
LH 145	28	31
LH 160	34	36.3
LH 289	30.5	32.8
Ep653	39.5	42.1
Ep505	59	61.8
L80	75	77.3
Ep450	27	30.2
Ep601	36.5	40.8
Ep624	42	46.7
LAM125	62	67.6
AM36	24	26.2
Ep450+G	21.5	30.1
L80+CF	57	61.2

**Table 3:** Results of ATD measurement: T_max_—maximum curing temperature; τ_gel_—gelation time.

Sample	T_max_ (°C)	τ_gel_ (min)
LH 145	218.5	31.0
LH 145+G	205.2	33.8
LH 145+CF	202.1	32.2
LH 160	197.8	36.3
LH 160+G	175.3	36.6
LH 160+CF	191.3	33.9
LH 289	211.0	32.8
LH 289+G	163.9	41.1
LH 289+CF	194.6	34.0
Ep450	85.6	30.2
Ep450+G	102.5	30.1
Ep450+CF	156.6	21.9
Ep505	120.7	61.8
Ep505+G	114.2	62.1
Ep505+CF	126.6	55.2
Ep601	213.4	40.8
Ep601+G	210.3	42.7
Ep601+CF	211.7	39.1
Ep624	208.3	46.7
Ep624+G	170.2	43.0
Ep624+CF	193.0	49.3
Ep653	201.8	42.1
Ep653+G	184.0	40.7
Ep653+CF	205.7	43.9
LAM125	191.3	67.6
LAM125+G	159.8	61.2
LAM125+CF	177.5	58.3
AM36	188.5	26.2
AM36+G	196.3	24.9
AM36+CF	228.3	25.7
L80	174.1	77.3
L80+G	147.9	73.5
L80+CF	172.5	61.2

## Data Availability

Not applicable.

## References

[1] Pielichowski J., Czub P., Penczek P., Bończa-Tomaszewski Z. (2016). Chemia i Technologia Żywic Epoksydowych.

[2] Oleksy M., Szwarc-Rzepka K., Heneczkowski M., Oliwa R., Jesionowski T. (2014). Epoxy Resin Composite Based on Functional Hybrid Fillers. Materials.

[3] Bréchet Y., Cavaillé J.Y., Chabert E., Chazeau L., Dendievel R., Flandin L., Gauthier C. (2001). Polymer Based Nanocomposites: Effect of Filler-Filler and Filler-Matrix Interactions. Adv. Eng. Mater..

[4] Chatys R. (2013). Investigation of the effect of distribution of the static strength on the fatigue failure of a layered composite by using the markov chain theory. Mech. Compos. Mater..

[5] Chatys R., Kleinhofs M., Panich A., Miśków G. Composite Laminates for Automotive Bumpers and Lightweight Support Structures. Proceedings of the 24th International Conference “Engineering Mechanics”.

[6] Huang H.-X., Zhang J.-J. (2009). Effects of filler-filler and polymer-filler interactions on rheological and mechanical properties of HDPE-wood composites. J. Appl. Polym. Sci..

[7] Chabert E., Bornert M., Bourgeat-Lami E., Cavaillé J.Y., Dendievel R., Gauthier C., Putaux J.L., Zaoui A. (2004). Filler–filler interactions and viscoelastic behavior of polymer nanocomposites. Mater. Sci. Eng. A.

[8] Bailly M., Kontopoulou M., El Mabrouk K. (2010). Effect of polymer/filler interactions on the structure and rheological properties of ethylene-octene copolymer/nanosilica composites. Polymer.

[9] Leziona J. (1998). Podstawy Technologii Kompozytów.

[10] Boczkowska A., Krzesiński G. (2016). Kompozyty i Techniki ich Wytwarzania.

[11] Shimkin A.A. (2016). Methods for the determination of the gel time of polymer resins and prepregs. Russ. J. Gen. Chem..

[12] (1999). ASTM D 2471–99: Standard Test Method for Gel Time and Peak Exothermic Temperature of Reacting Thermosetting Resins.

[13] IPC-TM-650 2 (1986). 3.18 Gel Time for Prepreg Materials.

[14] DOTD TR 703–85: Gel Time Determination of Epoxy Resin Systems. http://www.dotd.la.gov/Inside_LaDOTD/Divisions/Engineering/Materials_Lab/TPM_Vol_II_Part_VII/703.pdf.

[15] (2003). DIN EN ISO 2535:2003-02, Plastics—Unsaturated Polyester Resins—Measurement of Gel Time at Ambient Temperature.

[16] (1997). ISO 9396:1997 Plastics—Phenolic Resins—Determination of the Gel Time of Resols under Specific Conditions Using Automatic Apparatus.

[17] (2019). ASTM D3532/D3532M Standard Test Method for Gel Time of Carbon Fiber-Epoxy Prepreg.

[18] (1989). DIN 16945 Testing of Resins, Hardeners and Accelerators, and Catalyzed Resins.

[19] Laza J., Julian C., Larrauri E., Rodriguez M., Leon L. (1999). Thermal scanning rheometer analysis of curing kinetic of an epoxy resin: 2. An amine as curing agent. Polymer.

[20] Núñez-Regueira L., Gracia-Fernández C.A., Gómez-Barreiro S. (2005). Use of rheology, dielectric analysis and differential scan-ning calorimetry for gel time determination of a thermoset. Polymer.

[21] (2003). ASTM D4473—03Standard Test Method for Plastics: Dynamic Mechanical Properties: Cure Behavior.

[22] Beheshti M.H., Nasiri H., Vafaian M. (2005). Gel time and exotherm behaviour studies of an unsaturated polyester resin initiated and promoted with dual systems. Iran. Polym. J..

[23] Stark W. (2013). Investigation of the curing behaviour of carbon fibre epoxy prepreg by Dynamic Mechanical Analysis DMA. Polym. Test..

[24] Vassilikou-Dova A., Kalogeras I.M., Menczel J.D., Prime R.B. (2009). Thermal Analysis of Polymers: Fundamentals and Applications.

[25] Varley R.J., Hodgkin J.H., Hawthorne D.G., Simon G.P. (1996). Toughening of a trifunctional epoxy system. II. Thermal characterization of epoxy/amine cure. J. Appl. Polym. Sci..

[26] Gao J., Li L., Deng Y., Gao Z., Xu C., Zhang M. (1997). Study of gelation using differential scanning calorimetry (DSC). J. Therm. Anal. Calorim..

[27] Restrepo-Zapata N.C., Osswald T.A., Hernández-Ortiz J.P. (2014). Method for time–temperature–transformation diagrams using DSC data: Linseed aliphatic epoxy resin. J. Appl. Polym. Sci..

[28] Acitelli M., Prime R., Sacher E. (1971). Kinetics of epoxy cure: (1) The system bisphenol-A diglycidyl ether/m-phenylene diamine. Polymer.

[29] Lionetto F., Maffezzoli A. (2013). Monitoring the Cure State of Thermosetting Resins by Ultrasound. Materials.

[30] Seyhan A., Sun Z., Deitzel J., Tanoglu M., Heider D. (2009). Cure kinetics of vapor grown carbon nanofiber (VGCNF) modified epoxy resin suspensions and fracture toughness of their resulting nanocomposites. Mater. Chem. Phys..

[31] Chatys R., Piernik K. (2021). Influence pf speed of resin injection under pressure into mould on strength properties of polymer composite. Compos. Theory Pract..

[32] Koziol M., Mocek P., Jankowski P. (2016). Effect of the specimen volume of chemosetting polyester resin on the curing process. Polimery.

[33] Kozioł M. (2016). Nasycanie Ciśnieniowo-Próżniowe Zszywanych oraz Tkanych Trójwymiarowo Preform z Włókna Szklanego.

[34] Kozioł M., Gradoń P. (2018). Effect of glass fibre presence on curing process of unsaturated polyester resin. Compos. Theory Pract..

[35] Lou J., Zhang J., Xu S., Wang D., Fan X. (2021). New Method to Evaluate the Crosslinking Degree of Resin Finishing Agent with Cellulose Using Kjeldahl Method and Arrhenius Formula. Processes.

[36] Tcharkhtchi A., Nony F., Khelladi S., Fitoussi J., Farzaneh S. (2015). Epoxy/amine reactive systems for composites materials and their thermomechanical properties. Advances in Composites Manufacturing and Process Design.

[37] Jones P., Boontheung C., Hundt G. Employing an Arrhenius Rate Law to Predict the Lifetime of Oilfield Resins. Proceedings of the SPE International Conference on Oilfield Chemistry.

[38] Alonso M.V., Oliet M., García J., Rodríguez F., Echeverría J. (2006). Transformation of dynamic DSC results into isothermal data for the curing kinetics study of the resol resins. J. Therm. Anal. Calorim..

[39] Abraham S., Bodnar R., Lonnqvist J., Shahbazian F., Lagerstedt A., Andersson M. (2019). Investigation of Peritectic Behavior of Steel Using a Thermal Analysis Technique. Met. Mater. Trans. A.

[40] Tuttle R. (2021). Comparison of Rare Earth Refinement in 4130 and HY100. Metals.

[41] Binczyk F., Cwajna J., Gradoń P., Sozańska M., Cieśla M. (2013). Metallurgical Quality of Feed Ingots and Castings Made from Nickel and Cobalt Superalloys. Solid State Phenom..

[42] Migas D., Gradoń P., Mikuszewski T., Moskal G. (2020). Crystallization behavior of ternary γ–γ′ Co–Al–W alloy. J. Therm. Anal. Calorim..

[43] PPUH Z-TECH Zbigniew Jura. http://www.zjura.sownet.pl/z-tech/crystaldigraph.php.

[44] Rouwhorst J., Ness C., Stoyanov S., Zaccone A., Schall P. (2020). Nonequilibrium continuous phase transition in colloidal gelation with short-range attraction. Nat. Commun..

[45] Acocella M.R., Corcione C.E., Giuri A., Maggio M., Maffezzoli A., Guerra G. (2016). Graphene oxide as a catalyst for ring opening reactions in amine crosslinking of epoxy resins. RSC Adv..

[46] Corcione C.E., Acocella M.R., Giuri A., Maffezzoli A., Guerra G. (2015). Cure reaction of epoxy resins catalyzed by graphite-based nanofiller. AIP Conf. Proc..

[47] Corcione C.E., Acocella M.R., Giuri A., Maffezzoli A. (2016). Epoxy Resin Catalyzed by Graphite-Based Nanofillers. Int. Polym. Process..

[48] Huo S., Song P., Yu B., Ran S., Chevali V.S., Liu L., Fang Z., Wang H. (2021). Phosphorus-containing flame retardant epoxy thermosets: Recent advances and future perspectives. Prog. Polym. Sci..

[49] Huo S., Zhou Z., Jiang J., Sai T., Ran S., Fang Z., Song P., Wang H. (2022). Flame-retardant, transparent, mechanically-strong and tough epoxy resin enabled by high-efficiency multifunctional boron-based poly-phosphonamide. Chem. Eng. J..

[50] Epoxy and Polyester Resins, Hardeners and Gelcoats. https://www.havel-composites.com/en/categories/epoxy-and-polyester-resins--hardeners-and-gelcoats-363.

[51] Grupa Ciech S.A. (2019). Zakłady Organika Sarzyna. Katalog Żywice Epoksydowe i Utwardzacze Żywic Epoksydowych (Manufacturer’s Catalog).

